# Construction of a Diagnostic Model and Drug Prediction for Postischemic Stroke Cognitive Impairment Based on Machine Learning Screening of Lactate Metabolism– and Pyroptosis‐Related Genes

**DOI:** 10.1155/humu/2963117

**Published:** 2026-05-06

**Authors:** Shulong Ge, Qiying Zhang, Ning Liu, Xueyan Zheng, Han Xu, Li Zhang

**Affiliations:** ^1^ Department of Traditional Chinese Medicine, Shandong Provincial Third Hospital, Shandong University, Jinan, China, sdu.edu.cn; ^2^ Department of Internal Medicine, Jinan Municipal Government Hospital, Jinan, China; ^3^ General Outpatient Clinic, Jinan Second People′s Hospital, Jinan, China; ^4^ Department of Pharmacy, Jinan Second People′s Hospital, Jinan, China; ^5^ The First Clinical School of Medicine, Shandong University of Chinese Medicine, Jinan, China, sdutcm.edu.cn; ^6^ Department of Internal Medicine, Jinan Second People′s Hospital, Jinan, China

**Keywords:** bioinformatics, diagnostic biomarkers, lactate metabolism, machine learning, NLRP3 inflammasome, poststroke cognitive impairment, pyroptosis, single-cell sequencing

## Abstract

Reliable molecular biomarkers for poststroke cognitive impairment (PSCI) remain limited. Using publicly available bulk transcriptomic and single‐cell RNA‐seq datasets from GEO, we investigated lactate metabolism– and pyroptosis‐related signatures and developed a diagnostic model. Differential expression analysis, KEGG pathway enrichment, and weighted gene coexpression network analysis (WGCNA) were performed, followed by multialgorithm feature selection (LASSO, SVM‐RFE, and random forest). A logistic regression classifier was trained in the discovery cohort and externally validated in an independent cohort. Glycolysis/lactate metabolism, HIF‐1 signaling, and NOD‐like receptor‐related pathways were enriched in PSCI‐associated samples, and key coexpression modules were strongly correlated with ischemic injury traits. Cross‐model consensus identified LDHA, GSDMD, and CASP1 as hub genes, yielding an AUC of 0.912 (95% bootstrap CI: 0.841–0.983) in the training cohort and 0.885 (95% bootstrap CI: 0.798–0.972) in the validation cohort. Immune deconvolution and scRNA‐seq validation suggested increased proinflammatory microglia‐associated signals, with relatively higher LDHA expression in microglia than in neurons; cell–cell communication analysis highlighted inflammatory interactions including IL1B–IL1R1. Connectivity map (CMap) analysis nominated candidate compounds, and molecular docking predicted favorable binding between oxamate and LDHA (binding energy = −9.5 kcal/mol). Collectively, these findings propose a compact LDHA/GSDMD/CASP1 biomarker panel for PSCI diagnosis and provide hypothesis‐generating therapeutic leads that warrant further experimental validation.

## 1. Introduction

Stroke is one of the leading causes of death and long‐term disability worldwide, and approximately 70% of all stroke cases are ischemic in origin (“Corrigendum to: World Stroke Organization (WSO): Global Stroke Fact Sheet 2022” 2022). Poststroke cognitive impairment (PSCI) is one of the most common and severe sequelae of stroke, which can be summarized into a wide range of cognitive impairment, from mild cognitive impairment to dementia. Epidemiological research data show that about 20%–80% of stroke patients after the episode have varying degrees of cognitive dysfunction, and approximately 40% of them meet the diagnostic criteria for cognitive impairment within 1 year after stroke ([1, 2]). According to a scientific statement from the American Heart Association/American Stroke Association [3], PSCI seriously disables patients in terms of their activities of daily living, social engagement and quality of life, and has distinct negative impact on sustaining employment, being self‐sufficient and communicating with people. And the systematic review and meta‐analysis by Dowling et al. [4] also show that the patients with PSCI have a significantly higher risk of stroke recurrence and mortality. However, the underlying mechanisms of PSCI remain poorly understood, and reliable predictive biomarkers and targeted therapeutic strategies are still lacking.

Neuroinflammation and metabolic dysfunctions are believed to be important to the development of PSCI. At the metabolic level, the brain tissue is in the metabolism of a high energy demand but few reservoirs. After cerebral ischemia, due to impaired aerobic metabolism, cells can only use anaerobic glycolysis to produce ATP to sustain basic cellular activities ([5]). Lactate dehydrogenase A (LDHA) is a rate‐limiting enzyme in glycolysis that can reduce pyruvate to form lactic acid and takes the important step for energy metabolism under hypoxia. Jin et al. reported that elevated serum LDH levels after cerebral ischemia are associated with poor prognosis in ischemic stroke patients ([6]). More importantly, LDHA is not only involved in energy metabolism but also participates in the regulation of neuroinflammation by influencing microglial phenotypic polarization. Liu et al. [5] showed that LDHA inhibition suppresses the transition of microglia toward a proinflammatory phenotype and promotes a shift toward an anti‐inflammatory state, thereby exerting neuroprotective effects. In addition, Xiong et al. [7] reported that astrocyte‐derived lactate aggravates ischemic brain injury through protein lactylation, and astrocyte‐specific knockout of LDHA significantly reduced infarct size. Thus, LDHA‐induced dysregulation of the lactate metabolism may be an important pathological mechanism of ischemic brain injury.

Neuroinflammation and pyroptotic cell death have received increasing attention in recent years in the context of ischemic brain injury. Pyroptosis is a form of inflammatory programmed cell death that depends on gasdermin D (GSDMD) and is characterized by membrane pore formation, cell swelling, membrane rupture, and release of proinflammatory cytokines ([8]). The NLRP3 inflammasome is a molecular platform that mediates the pyroptosis, it comprises the pattern recognition receptor NLRP3, the adaptor protein ASC, and the effector pro–caspase‐1. Huang et al. [9] provided a more comprehensive illustration of the activation process: Upon activation, NLRP3 can cause caspase‐1 to get activated as well, which makes cleaved GSDMD into membrane pores while promoting their processing and releasing IL‐1Beta and IL‐18, thus triggering a sharp inflammation. In ischemic brain injury, Long et al. [10] systematically summarized multiple signal pathways by which the NLRP3 inflammasome activates pyroptosis, such as the TLR4/NF‐*κ*B/NLRP3 and ROS/TXNIP/NLRP3 pathways. And Liu show that chemr23 signal activates can reduce brain injuries total size because it causes brain cells pyroptosis caused by inhibition of NLRP3 Inflammasome, as mentioned before ([11]). Cognitive function with CX3CR1 deletion that promotes cognitive recovery from stroke by inhibiting microglia pyroptosis‐mediated neuroinflammation according to Ge et al. [12]. Xu et al. [13], in a recent review, pointed out that neuroinflammation caused by the activation of NLRP3 inflammasome is the core pathogenic mechanism in stroke and other neurological diseases.

Importantly, lactate metabolism and pyroptosis are closely interconnected. Dysregulated glycolytic flux may act as a metabolic signal that promotes NLRP3 inflammasome activation and subsequently induces pyroptotic cell death through mitochondrial ROS signaling [14]. F. X. Wang et al. [15, 16] also showed that accumulated lactate could also regulate its expression level by self‐transcriptional level under pyroptotic histone lactylation, and lactylation also had obvious efficacy for I‐R injury treatment. And then, it was a study by Li so forth, that just recently displayed that what takes place with reprogramming of metabolism in an ischemic stroke is an acute glycolysis crisis and inhibition of LDHA is also able to prevent the ultra‐acute acidosis as well as the pool of succinate that is found in a microglia, which has the ability to activate NLRP3 [15, 16]. The collective result is that lactate metabolism–pyroptosis axis might be a regulatory network in the development and progression of PSCI.

Although previous studies have explored the mechanisms of ischemic brain injury from the perspectives of lactate metabolism or pyroptosis, respectively, the systematic integration of the two biological processes to identify diagnostic biomarkers and therapeutic targets for PSCI is still lacking. Therefore, the present study has combined the transcriptomic sequencing data and single‐cell RNA sequencing data and uses weighted gene co‐expression network analysis (WGCNA) and multiple machine learning algorithms (Least Absolute Shrinkage and Selection Operator [LASSO], Support vector machine–recursive feature elimination [SVM‐RFSE], and random forest) to systematically screen out the core genes related to lactate metabolism and pyroptosis. A diagnostic prediction model for PSCI is established, and the functions of the core genes and possible therapeutic drugs are validated through immune infiltration analysis, analysis of immune cells–cell communication and molecular docking, and the aim of providing new biomarkers and candidate targets for the early diagnosis and precise treatment of PSCI.

## 2. Materials and Methods

### 2.1. Data Sources and Preprocessing

All transcriptomic data used in this study was downloaded from the Gene Expression Omnibus (GEO, https://www.ncbi.nlm.nih.gov/geo/) [17]. Three sets of data associated with PSCI were retrieved and downloaded (Table S1): GSE223580 (Illumina NovaSeq platform, which includes hippocampal tissue samples from three mice subjected to a sham operation and three bilateral common carotid artery stenosis [BCAS]) was taken as the discovery cohort; GSE137482 (Illumina NextSeq platform, which contains parietal cortex samples from six mice and six middle cerebral artery occlusion [MCAO] model mice) was utilized as an independent validation cohort; GSE174574 (10× Genomics platform, which consists of single‐cell RNA sequencing data from the ischemic hemispheres of three mice with a sham operation and three MCAO model mice) was used for single‐cell‐level validation analysis.

After downloading the raw data, preprocessing was carried out with R software (Version 4.5.0). For bulk RNA‐seq data, background correction and quantile normalization were carried out with the limma package [18]. Genes that were not expressed (expression value less than 1 in all samples) were not selected, and the expression matrix was converted to log2. Use ComBat to correct for batch effects [19]. Principal component analysis (PCA) and intersample correlation heat maps were used for quality assessment.

### 2.2. Construction of Lactate Metabolism–Pyroptosis‐Related Gene Set

Gene sets related to lactate metabolism and pyroptosis were curated from the Molecular Signatures Database (MSigDB, https://www.gsea-msigdb.org/gsea/msigdb/) [20]. Specifically, HALLMARK_GLYCOLYSIS (MSigDB ID: M5937) was used to define lactate metabolism‐related genes, including LDHA, LDHB, SLC16A1 (MCT1), SLC16A3 (MCT4), PDK1, PKM2, and HK2. REACTOME_PYROPTOSIS (MSigDB ID: M27580) was used to define core pyroptosis‐related genes, including GSDMD, GSDME, CASP1, CASP4, CASP5, NLRP3, PYCARD (ASC), AIM2, IL1B, and IL18. In addition, seven cross‐regulatory node genes (HIF1A, HMGB1, TXNIP, NFKB1, SIRT2, STAT3, and TLR4) were included based on literature curation because of their reported roles in linking metabolic stress and inflammasome activation. In total, 24 genes were included in the subsequent intersection analysis. This gene panel was defined prior to differential expression analysis to avoid circular reasoning and selection bias. The complete gene list and inclusion rationale are provided in Table S6.

### 2.3. Differential Expression Analysis and Functional Enrichment

Differential expression analysis was carried out in the discovery cohort using the limma package. Absolute log2 fold change greater than 1, adjusted *p* value less than 0.05 were regarded as differentially expressed genes (DEGs). The results of the differential analysis were visualized as volcano plots and heat maps. And through the use of the clusterProfiler package to perform KEGG pathway enrichment analysis on the DEGs ([21]; G. [22]). Adjusted *p* value less than 0.05 was considered to be significantly enriched pathways. Enrichment results were visualized using bubble plots, in which bubble size represented gene count and color intensity reflected the adjusted *p* value.

### 2.4. WGCNA

WGCNA was carried out to generate a gene coexpression network and find gene modules strongly related to the PCSI phenotype [23]. First of all, clustering of samples was carried out, and then the outliers were removed. A soft thresholding power of *β* = 7 was chosen so that the network would meet the scale‐free topology criterion (scale‐free topology model fit > 0.85). Gene modules were identified with a dynamic tree‐cutting algorithm and a minimum module size of 30 genes.

Module eigengenes (MEs) were calculated, and Pearson correlation analysis was performed between MEs and phenotypic traits, including ischemia duration and cognitive score, to identify modules significantly associated with PSCI‐related traits. Key modules′ genes in terms of modules memberships (MM) and gene significances (GS). Core genes within key modules were defined as those with MM > 0.8 and GS > 0.5.

DEGs, the lactate metabolism–pyroptosis‐related gene set, and genes from the WGCNA key modules were intersected to get a set of candidate genes for the following machine learning analyses.

### 2.5. Machine Learning–Based Screening of Core Genes

Three types of machine learning algorithms were used on the candidate genes as features for a feature selection approach in order to select out the core genes that have the greatest diagnostic value.

LASSO regression was executed through the glmnet package. Tenfold cross‐validation was used to determine the optimal penalty parameter lambda, and lambda.1se was selected to identify genes with nonzero coefficients.

The SVM‐RFE was performed using the e1071 package. Features were recursively eliminated while evaluating model performance to identify the best feature set. Fivefold cross‐validation was used to evaluate the classification accuracy at different numbers of features.

Random forest analysis was carried out using the random forest package. A random forest model of 500 trees was built, and gene importance was evaluated according to the standard of Gini importance. The Top 10 important genes were selected based on the importance.

Intersect the genes identified by all three algorithms to determine the final hub genes. In the independent validation cohort (GSE137482), the differential expression of the hub genes was done by using the Wilcoxon rank sum test.

### 2.6. Construction and Evaluation of the Diagnosis Model

According to the screened hub genes, we establish the diagnostic prediction model of PSCI based on the logistic regression approach. GSE223580 was used as the training set to establish the model, and GSE137482 was used as the independent validation set to validate the generalization performance of the model.

The diagnostic performance of the model was evaluated using ROC curve analysis, and the area under the curve (AUC) was calculated. Bootstrap 95% confidence intervals (CIs) were computed using 2000 stratified resamples implemented in the pROC package, using the ci.auc function with the bootstrap method (boot.n = 2000). The analysis was performed in R using the ci.auc function with the bootstrap method (boot.n = 2000). A nomogram was generated via the rms package for the model with the graphics to demonstrate the model to help the clinical application.

It was observed if calibration looked good on a calibration curve examining if the predicted probabilities match the actual results. Clinical utility of the model has been measured through Decision curve analyses (DCAs) that are performed for estimating the benefit from using the model over a range of threshold probabilities [24]. In addition, we generated the clinical impact curve for the prediction results at different risk thresholds.

### 2.7. Immune Infiltration Analysis

The CIBERSORT algorithm was used to estimate immune‐related transcriptional signatures in the samples [25]. CIBERSORT is a deconvolution algorithm based on support vector regression that infers the relative abundance of immune‐related signatures from bulk transcriptomic profiles. In the present study, the LM22 signature matrix was used for CIBERSORT analysis. Because LM22 was originally developed for peripheral leukocyte populations and does not explicitly include brain‐resident cell types such as microglia, the resulting deconvolution estimates should be interpreted with caution. To further assess robustness, MCP‐counter analysis was additionally performed, and the directional findings were qualitatively consistent (Figure S1). Spearman correlation analysis was used to evaluate the associations between hub gene expression and inferred immune‐related signatures. The results were visualized as lollipop plots, in which dot size represented the absolute correlation coefficient and color intensity reflected statistical significance.

### 2.8. Single‐Cell RNA‐Seq Data Analysis

Single‐cell RNA sequencing data from the GSE174574 dataset were obtained from the GEO database, and the analysis was carried out using Seurat (Version 4.0) [26]. The quality control criteria were as follows: 200–5000 detected genes and a mitochondrial gene ratio lower than 20%. Data normalization and highly variable gene identification were carried out with the SCTransform method [27].

PCA was performed for dimensionality reduction, and the first 30 principal components were used in the subsequent analysis. Cell clustering was carried out by the FindNeighbors and FindClusters functions with a resolution parameter of 0.5, and then the UMAP algorithm was used for cluster visualization. Cell types were annotated based on previously described marker genes such as astrocytes (GFAP and AQP4), microglia (CX3CR1 and ITGAM), and neurons (RBFOX3 and SYP).

The expression distribution of hub genes in different cell types was visualized by FeaturePlot and VlnPlot. Although clusters consistent with oligodendrocytes and endothelial cells were also identified in the dataset, the primary analysis focused on astrocytes, microglia, and neurons because these cell types represent the major neuroinflammatory and neuron–glia interaction compartments in ischemic brain injury. For completeness, hub gene expression across all annotated cell clusters is provided in Figure S2.

### 2.9. Cell–Cell Communication Analysis

CellChat was used to explore the cell type to cell type communication networks (S. [28]). CellChat combines with the ligand–receptor interaction database to quantify how strong and likely the signal transmission between cells.

Then, a CellChat object was created through the import of single‐cell expression data and cell type annotation information. Identified overexpressed ligands and receptors using the identifyOverExpressedGenes and identifyOverExpressedInteractions functions. And computed as well as among numerous kinds of cells also by shuffling determined stats probabilities were made.

Communication results were visualized using a circular plot, and we used the signal strength of the cell types each cell was a signal sender or receiver. And also, when it comes to communication path ways with the HUB GENES: We did an analysis on each individual pair of ligand‐reolocue as well as create a bubble plot, will there be some way so that we may communicate? As well as to determine whether or not it will be statistically significant.

### 2.10. Candidate Drug Prediction

The Connectivity map (CMap; https://clue.io/) database was used to predict candidate compounds that could reverse PSCI‐associated transcriptional signatures [29]. CMap finds small‐molecule compounds that may have therapeutic value by comparing drug‐induced gene expression patterns to disease‐specific gene expression patterns.

Hub genes were submitted to the CMap platform as the disease signature gene set, and connectivity scores were retrieved. Negative connectivity values indicate that a compound can reverse the gene expression changes associated with the disease. A connectivity score below −0.9 was used as a stringent screening criterion to prioritize compounds with the strongest inverse transcriptional signatures. This threshold is consistent with the L1000 CMap framework and previous CMap‐based drug repurposing studies, in which |*N* *C* *S*| > 0.90 is considered indicative of strong biologically meaningful connectivity [29, 30]. Compounds with connectivity scores between −0.85 and −0.90 were retained as secondary candidates in Table S5B.

### 2.11. Molecular Docking Validation

Molecular docking was carried out by means of AutoDock software (Version 4.2.6) to confirm the binding kinetic energy of the potential drug molecules and target proteins [31]. Three‐dimensional structures of the target proteins were acquired from Protein Data Bank (PDB, https://www.rcsb.org/) [32]. Three‐dimensional structures of the candidate compounds were downloaded from PubChem database (https://pubchem.ncbi.nlm.nih.gov/)“https://pubchem” and converted to the right formats with Open Babel [33, 34].

Before docking, water molecules and original ligands were deleted from the protein structure, polar hydrogen atoms, and Kollman charges were added using AutoGrid to set the grid box parameters to cover the active site. Docking calculations were carried out using a Lamarckian genetic algorithm; 100 individual docking calculations were run for each ligand–protein.

Docking results were judged by the binding energy (kcal/mol) of the docking results, and a more negative value indicated better docking affinity. Bound energies less than −7.0 kcal/mol were taken to indicate strong binding of ligands to target. The docking conformations and analyze the main interacting residues and interaction types were visualized by PyMOL software.

### 2.12. Statistical Analysis

All data analysis was carried out on the R software (Version 4.5.0). Comparisons among 2 groups were done using the Wilcoxon rank‐sum test or Student′s *t*‐test, whereas comparing among 3+ groups the Kruskal–Wallis test was performed. Correlation analyses were done with Pearson or Spearman correlation coefficients. *p* value < 0.5 was considered as statistically significant. Data visualization was mainly carried out with the ggplot2 package.

## 3. Results

### 3.1. Data Preprocessing and DEG Analysis

**Figure 1:**
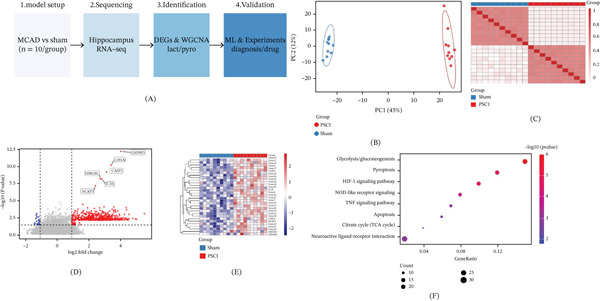
Data preparation and differential expression analysis. (A) Study workflow. Publicly available GEO datasets were analyzed, including GSE223580 as the discovery cohort (hippocampal tissue from three sham and three BCAS mice), GSE137482 as the bulk validation cohort, and GSE174574 as the single‐cell validation cohort. (B) PCA plot showing separate clusters between PSCI and sham. PC1 and PC2 explained 45% and 12% of the variance, respectively. (C) Sample correlation heat map showing high internal consistency. (D) Volcano plot of differentially expressed genes. Key lactate metabolism and pyroptosis‐related genes (LDHA, GSDMD, CASP1, NLRP3, IL1B, and HMGB1) are marked. (E) Heat map of lactate metabolism and pyroptosis signature genes expression of samples. (F) KEGG pathway enrichment analysis of DEGs. Significantly enriched pathways were glycolysis/gluconeogenesis, pyroptosis, HIF‐1 signaling pathway, and NOD‐like receptor signaling pathway.

The entire analytical process of this study is shown in Figure 1A. Transcriptomic datasets that are associated with PSCI were got from the GEO database (Table S1); among them, we chose GSE223580 for the discovery cohort and used GSE137482 and GSE174574, respectively, for the validation cohorts, including both single‐cell‐level and bulk‐level validation.

PCA of the discovery cohort identified a clear distinction between the PSCI group and the sham group based on the PC1 dimension, with 45% of the variance explained, and the PC2 axis explaining an additional 12% of the variance (Figure 1B), suggesting that the two groups of samples had significant transcriptomic differences. The intersample correlation heat map also confirmed that the groups have a high degree of consistency and can be clearly distinguished between groups (Figure 1C).

With |log2FC| > 1 and *p* < 0.05 as the criteria, a group of DEGs was selected. Volcano plot showed that there were many genes related to lactate metabolism and pyroptosis, which were significantly upregulated in the PSCI group, such as LDHA, GSDMD, CASP1, HMGB1, NLRP3, and IL1B (Figure 1D). Clustering analysis of the lactate metabolism–pyroptosis‐related gene set revealed that these genes showed strong discriminatory ability between the PSCI and sham groups, as shown in the heat map (Figure 1E).

KEGG pathway enrichment analysis showed that the DEGs were significantly enriched in glycolysis/gluconeogenesis (*p* < 0.001), pyroptosis, HIF‐1 signaling pathway, NOD‐like receptor signaling pathway, TNF signaling pathway, and so on (Figure 1F, Table S2). Among which, there were 25 enriched genes in the glycolysis pathway and 20 enriched genes in the NOD‐like receptor signaling pathway, suggesting that lactate metabolism and pyroptotic processes may play important roles in the pathogenesis of PSCI.

### 3.2. Identification of PSCI‐Related Key Gene Modules by WGCNA

**Figure 2:**
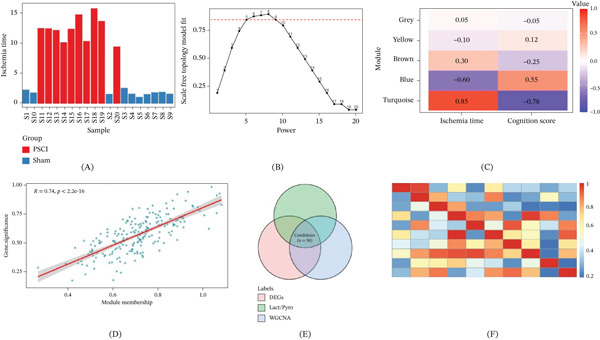
WGCNA analysis and candidate genes. (A) The sample clustering dendrogram with clinical traits. Bar plot shows ischemia time of each sample, the PSCI group (red) has a longer ischemia time than the sham group (blue). (B) Scale‐free topology model fit across the soft threshold powers. The red dashed line is the R^2^ of 0.85. (C) Module–trait relationship heat map. Turquoise module displays the strongest positive correlation with ischemia time *r* = 0.85 and negative correlation with cognition score (*r* = −0.78). (D) Scatterplot of module membership versus gene significance for turquoise module (*R* = 0.74, *p* < 2.2*e* − 16). (E) Venn diagram representing the intersection of differentially expressed genes, lactate metabolism/pyroptosis‐related genes, and WGCNA turquoise module genes, resulting in 58 candidate genes. (F) Topological overlap matrix (TOM) heat map indicating gene coexpression network connectivity.

To further find out the gene modules closely related to PSCI, WGCNA was carried out. From the sample clustering, it could be observed that all the samples passed the quality control, and the ischemia time of the PSCI group was significantly longer than that of the sham group (Figure 2A).

In the soft‐threshold selection process, when *β* = 7, the scale‐free topology model fit index was greater than 0.85 (Figure 2B), and it met the requirements for constructing a scale‐free network. According to this parameter, we distinguished five gene coexpression modules that are labeled in different colors (Table S3).

Module–trait relationship analysis showed that the Turquoise module was strongly and positively related to ischemia duration (*r* = 0.85, *p* < 0.001) and was also negatively correlated with cognitive scores (*r* = −0.78, *p* < 0.001), indicating that the genes in this module were closely related to PSCI (Figure 2C). Whereas the blue module showed an inverse correlation pattern and was negatively correlated with the duration of ischemia (*r* = −0.60) and positively correlated with cognitive scores (*r* = 0.55), the brown, yellow, and gray modules were not significantly correlated with phenotypic traits.

The scatterplot of MM versus GS within the Turquoise module showed a strong positive correlation (*r* = 0.74, *p* < 2.2 × 10^−16^), further supporting the biological relevance of this module to PSCI‐related traits (Figure 2D).

To obtain biologically meaningful yet data‐supported candidate genes, we intersected three gene sets: DEGs in the discovery cohort, the curated lactate metabolism/pyroptosis‐related gene panel, and genes from the PSCI‐associated WGCNA key module. This step yielded 58 candidate genes (Figure 2E), which were then subjected to machine learning–based feature selection. TOM heat map showed the coexpression relationship among those candidate genes (Figure 2F).

### 3.3. Machine Learning–Based Identification of Core Diagnostic Genes

To screen out the most valuable core genes among the 58 candidate genes, 3 machine learning algorithms were used.

**Figure 3:**
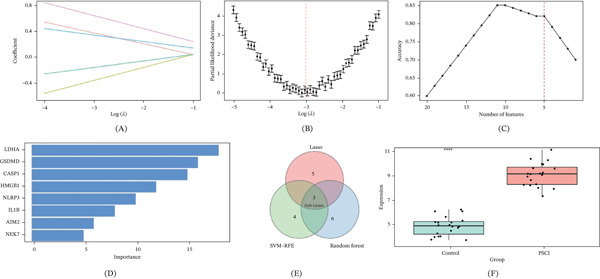
Machine learning–based hub gene identification. (A) The coefficient profiles of the candidates of hub genes at different lambda values. (B) LASSO cross‐validation plot for optimal lambda selection based on partial likelihood deviance. (C) SVM‐RFE accuracy curve, which displays the model performance with the descending number of features. (D) Random forest variable importance ranking. LDHA, GSDMD, and CASP1 had the highest importance scores. (E) Venn diagram of the genes chosen by LASSO, SVM‐RFE, and random forest algorithms. Three hub genes (LDHA, GSDMD, and CASP1) at the intersections. (F) LDHA expression difference between the control and PSCI groups ∗∗∗∗*p* < 0.0001.

According to the results of LASSO regression analysis, with the increase of the penalty factor *λ*, most gene coefficients shrink toward zero (Figure 3A). Tenfold cross‐validation was used to find the best *λ* (Figure 3B), select the top eight genes with nonzero coefficients.

SVM‐RFE analysis indicated that the highest classification accuracy, around 0.82, was obtained with a number of features between three and five (Figure 3C). More reduction in the amount of features resulted in a drop in accuracy.

Random forest analysis determined the gene importance with Gini index; it could be found that top five genes including LDHA, GSDMD, CASP1, HMGB1, and NLRP3 both had high value in terms of Gini importance (Figure 3D).

Intersecting the gene sets identified by the three algorithms yielded three final hub genes: LDHA, GSDMD, and CASP1 (Figure 3E; Table S4). These genes represent key nodes of lactate metabolism (LDHA) and pyroptosis (GSDMD and CASP1). In the independent validation cohort, LDHA expression was significantly higher in the PSCI group than in the control group (*p* < 0.0001), further supporting its potential diagnostic value (Figure 3F).

### 3.4. Construction and Evaluation of the Diagnosis Model

**Figure 4:**
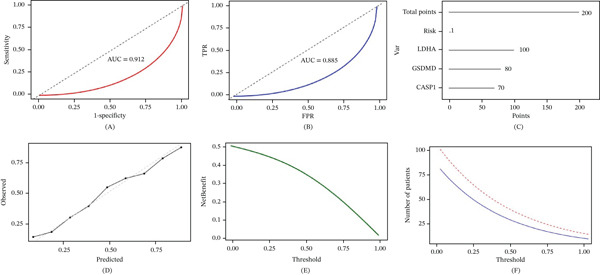
Diagnostic model construction and evaluation. (A) ROC curve of the diagnostic model in the training set (AUC = 0.912, 95% bootstrap CI: 0.841–0.983). (B) ROC curve of the diagnostic model in the independent validation set (AUC = 0.885, 95% bootstrap CI: 0.798–0.972). (C) Nomogram for predicting PSCI risk based on the expression levels of LDHA, GSDMD, and CASP1. (D) Calibration curve indicating agreement between predicted and observed probabilities. (E) Decision curve analysis (DCA) showing the net clinical benefit of the diagnostic model across different threshold probabilities. (F) Clinical impact curve showing the number of samples classified as high risk at different threshold probabilities.

According to the three hub genes, a diagnostic model for PSCI was constructed. The model achieved an AUC of 0.912 (95% bootstrap CI: 0.841–0.983) in the training cohort (Figure 4A) and 0.885 (95% bootstrap CI: 0.798–0.972) in the validation cohort (Figure 4B). These results indicate good model discrimination despite the relatively small sample size. For clinical applicability, the model was further visualized as a nomogram (Figure 4C). Clinicians give corresponding scores according to the expression levels of CASP1, GSDMD, and LDHA, add up the scores to get a total score, and then estimate the probability of occurrence of PSCI.

The calibration curve showed good agreement between the predicted and observed probabilities, indicating satisfactory calibration of the diagnostic model (Figure 4D).

DCA to evaluate the model clinical utility as seen in Figure 4E. The model gave a positive net benefit across a wide array of threshold probabilities and performed better than both treat‐all and treat‐none. The clinical impact curve further verified the predictive ability of the model at different risk threshold levels (Figure 4F).

### 3.5. Immune Infiltration and Single–Cell‐Level Validation

**Figure 5:**
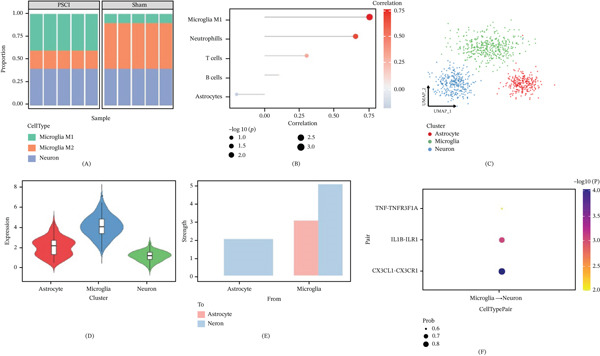
Immune infiltration and single‐cell RNA‐seq. (A) Stacked bar plot of immune‐related signature composition in PSCI and sham samples. PSCI samples showed increased proinflammatory microglia‐like signatures. (B) Correlation analysis between LDHA expression and immune cell infiltration. LDHA is positively associated with M1‐like microglial signatures. (C) The UMAP visualization based on single‐cell RNA sequences (GSE174574) for the three types of cells used in this analysis—astrocytes, microglia, and neuron. (D) Violin plot showing LDHA expression levels across annotated cell types. Microglia showed the highest LDHA expression. (E) Global analysis of intercellular communication strength showing that microglia exhibited prominent outgoing signaling toward other cell populations. (F) Bubble plot of key ligand–receptor interactions between microglia and neurons, including CX3CL1–CX3CR1, IL1B–IL1R1, and TNF–TNFRSF1A.

Explore the relationship between hub genes and the immune microenvironment; immune cell infiltration analysis is carried out. The immune deconvolution analysis suggested an increase in proinflammatory microglia‐like signatures and a decrease in anti‐inflammatory microglia‐like signatures in PSCI samples compared with the sham group (Figure 5A), suggesting that PSCI is accompanied by the activation of proinflammatory immune cells.

Correlation analysis shows that LDHA expression was positively correlated with the degree of M1‐type microglial infiltration (*r* > 0.75, *p* < 0.001). LDHA expression was also positively correlated with neutrophils and T cells, but it was correlated with B cells and astrocytes to a lower degree (Figure 5B).

Single‐cell RNA sequencing data GSE174574 was used to verify. Figure 5C shows UMAP‐based dimensionality reduction and clustering classified cells in three main populations: astrocytes, microglia, and neurons. LDHA showed the highest expression in microglia, then in astrocytes, and had a relatively low expression in neurons (Figure 5D).

Analysis of cell–cell communication showed that microglia had a lot of signal output to neurons and astrocytes (Figure 5E). Further analysis of the ligand–receptor interaction pairs of microglia and neurons revealed that CX3CL1–CX3CR1, IL1B–IL1R1, and TNF–TNFRSF1A were the major communication pathways (Figure 5F). These were mostly involved in inflammatory signaling and were very close to the release of inflammatory mediators after pyroptosis was activated.

### 3.6. Prediction of Potential Therapeutic Drugs and Molecular Docking Verification

**Figure 6:**
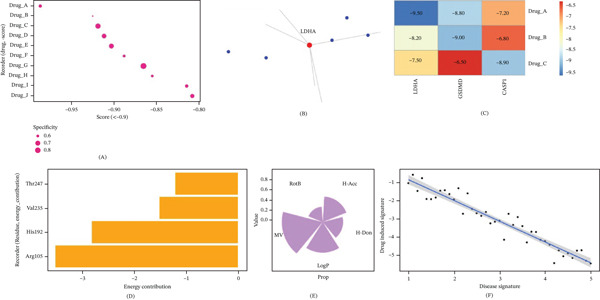
Drug prediction and molecular docking analysis. (A) CMap connectivity scores for Top 10 candidates. Drugs with scores smaller than −0.9 were deemed possible therapeutic drugs that can restore PSCI gene signature. (B) Drug–target interaction network illustrating the connection between candidate drugs and hub genes. (C) Molecular docking binding energy heat map of candidate compounds against hub gene‐related target proteins. More negative binding energy indicates stronger predicted binding affinity. (D) Key binding‐residue analysis for the oxamate‐LDHA complex (oxamate, PubChem CID: 971; CAS: 996‐31‐6). Arg105, His192, Val235, and Thr247 contributed to the predicted binding interaction. (E) Physicochemical properties of drug candidates include hydrogen bond acceptors (H‐Acc), hydrogen bond donors (H‐Don), LogP, molecular weight (MW), and rotatable bonds (RotB). (F) Gene signature reversal analysis indicates drug‐induced signatures negatively correlated with disease signatures.

Based on the expression characteristics of the hub genes, CMap database was used to find out the drug candidates that could reverse the molecular signature of PSCI. By setting the connectivity score < −0.9 as the screening condition, 10 small‐molecule compounds with possible therapeutic effects were identified (Figure 6A).

By building a drug–target interaction network, it can be seen that the screened candidate compounds mainly targeted LDHA, CASP1, and GSDMD (Figure 6B). Among the identified compounds, oxamate showed the strongest predicted association with LDHA. Molecular docking analysis further indicated that oxamate had the lowest binding energy with LDHA (−9.5 kcal/mol), whereas VX‐765 (Belnacasan) and disulfiram showed favorable predicted binding to CASP1 (−8.9 kcal/mol) and GSDMD (−8.2 kcal/mol), respectively (Figure 6C; Table S5). In the oxamate–LDHA complex, the main binding‐site residues included Arg105, His192, Val235, and Thr247, among which Arg105 contributed prominently to binding (Figure 6D).

In addition, physicochemical property analysis showed that the selected candidate compounds generally satisfied basic drug‐likeness criteria, including molecular weight, hydrogen bond donor/acceptor counts, LogP, and rotatable bond number (Figure 6E). Gene signature reversal analysis further supported that these compounds exhibited inverse transcriptional associations with the PSCI‐related hub gene signature (Figure 6F).

## 4. Discussion

The pathogenesis of PSCI is a complex interaction of multiple pathophysiological processes, but the molecular regulatory network of PSCI is still not fully elucidated. [3]. From the integrative view of lactate metabolism and pyroptosis, this study found LDHA, GSDMD, and CASP1 to be core diagnostic genes for PSCI via multiomics integration and built a diagnostic model with good predictive performance. These findings provide new ideas for the research of the molecular pathogenesis of PSCI.

The present study employed two mechanistically distinct ischaemia models: the BCAS model (chronic cerebral hypoperfusion, GSE223580) and the MCAO model (acute focal ischaemia, GSE137482). Although these models differ in temporal dynamics and affected brain regions, this heterogeneity enhances translational robustness. Biomarkers consistently dysregulated across both paradigms are less likely to represent model‐specific artifacts and more likely to reflect core molecular features of PSCI.

KEGG enrichment analysis showed that DEGs were significantly enriched in glycolysis, the HIF‐1 signaling pathway and the NOD‐like receptor signaling pathway, indicating that metabolic reprogramming and inflammatory cell death jointly participate in the pathogenesis of PSCI. When there is an ischemic and hypoxic situation, the HIF‐1*α* is activated to upregulate the key glycolytic enzyme, which produces an excessive accumulation of lactate (Y. [5]). Beyond its metabolic role, lactate may also regulate inflammatory gene transcription through histone lactylation [7, 35] although this mechanism was not directly investigated in the present study. These enrichment results are also rational to focus on the lactate metabolism–pyroptosis axis and will be to find and judge the next hub genes.

Based on the above findings, WGCNA was carried out to identify gene modules closely correlated with PSCI phenotypes. For the Turquoise module, it was found that there was a strong positive relationship with ischemia duration (*r* = 0.85) and a negative relationship with cognitive scores (*r* = −0.78), which means the expression changes in this module are temporally aligned with PSCI progressing and the cognitive deterioration. Intersection of the module genes and the lactate metabolism–pyroptosis gene set provided 58 candidate genes that were then further filtered using the LASSO, SVM–RFE, and random forest algorithms. Agreement on LDHA, GSDMD, and CASP1 by multiple algorithms enhanced the robustness of hub genes [30] and suggested that such genes were at the core of the lactate metabolism–pyroptosis regulatory network.

Among the three hub genes, LDHA has a more prominent function. As an important enzyme in the junction of glycolysis and lactate metabolism, the expression level of LDHA at an elevated level in PSCI model and strongly associated with M1‐type microglia (*r* > 0.75) indicate a possible way to achieve metabolic‐inflammation cross talk. Microglia, the resident immune cells of the central nervous system, and the polarization status is the key to determining the behavior of neuroinflammation [36]. M1 type microglia depend mainly on glycolysis for energy and increased LDHA activity would be necessary to support the metabolic requirements for a proinflammatory phenotype [15, 16]. Xiong et al. [7] discovered that the laactate from producing by astrocyte will be hurt by protein‐lactation and neuroma cells by stopping LDHA then the volume of the infarct can reduce. In the present study, through single‐cell analysis, it can be found that LDHA expression is highest in microglia, and combined with immune infiltration results, these results indicate that LDHA may help maintain a proinflammatory metabolic state in microglia to participate in PSCI.

On the contrary of LDHA′s metabolic regulatory function is the concurrent discovery of GSDMD and CASP1, the central pathway of the pyroptotic execution for PSCI. CASP1, as the effector enzyme of inflammasome cleaves the GSDMD, leading to the release of its pore‐forming N‐terminal domain, eventually causing cell membrane rupture and inflammatory cytokines to be released. CASP1 and GSDMD make up the core execution cascade of pyroptosis (Y. [9]). Both genes′ expression levels in this experiment were related to the severity of PSCI and positively correlated with each other, in line with the activation hierarchy of the pyroptosis pathway. As for the previous study, it has confirmed that CX3CR1 knockout improves poststroke cognitive function through inhibiting microglial pyroptosis [12], as well as that activation of ChemR23 exhibits neuroprotection by inhibiting the NLRP3/CASP1/GSDMD pathway (L. [11]), offering additional experimental evidence for present findings.

The functional effects of these hub genes depend on particular intercellular communication networks. From the analysis of cell–cell communication, it can be known that the main ligand–receptor pair mediating neuron–microglia interaction was CX3CL1–CX3CR1, IL1B–IL1R1, and TNF–TNFRSF1A. CX3CL1–CX3CR1 under physiologic state to maintain microglial quiescence, but dysregulated by pathological condition promotes microglial activation and pyroptosis [12]. Altered communication strength of this axis in the PSCI model indicates disrupted neuron–microglia homeostatic signaling. IL1B–IL1R1 signaling has been enhanced, CASP1 activates IL‐1*β* maturation directly, and it is a key downstream inflammatory pathway of pyroptosis. Such communication merges LDHA‐mediated metabolic disarray with GSDMD/CASP1‐driven pyroptosis into one pathological circuit.

The diagnostic model constructed based on the three hub genes still had good predictive performance in the independent validation cohort (AUC = 0.885), and the calibration and DCA also provided support for its clinical application. Compared with previous models based on clinical or image features [4], the molecular biomarker‐based model is more biological and all core genes converge on the lactate metabolism–pyroptosis axis [30]. This also improves the biological interpretability of the model. It is necessary to overcome the consistency between brain tissue expression and peripheral biomarkers in clinical translation, and future studies can investigate the feasibility of serum LDHA as a surrogate marker.

In addition to risk stratification, CMap‐based drug prediction combined with molecular docking provides preliminary insights into potential therapeutic strategies. Candidate compounds had binding energy lower than −7.0 kcal/mol toward LDHA, CASP1, and GSDMD, suggesting target engagement. Previous studies have pointed out the potential of NLRP3 inhibitors such as MCC950 in therapeutic applications in neurological disease models [13], and small‐molecule LDHA inhibitors have also been discovered in a cancer metabolism research context [15, 16]. Expanding on these strategies for PSCI has translational potential, but questions such as BBB permeability and specificity to the brain need to be considered carefully.

Several limitations of this study should be acknowledged. First, the sample sizes of the discovery and validation cohorts were relatively small, which may affect the robustness and stability of the model. Second, the discovery (BCAS) and validation (MCAO) cohorts differ in model mechanism, brain region, and temporal profile. Therefore, cross‐cohort consistency should be interpreted as evidence of generalizability rather than pathophysiological equivalence. A methodological limitation concerns the use of the LM22 signature matrix, which was originally developed for peripheral blood leukocytes and does not include brain‐resident cell types as defined cellular references. Although the relative increase in proinflammatory microglia‐associated signatures was corroborated by single‐cell RNA‐seq data and qualitatively replicated with MCP‐counter analysis, absolute proportion estimates derived from CIBERSORT/LM22 should be interpreted with caution. Future studies using brain tissue–validated deconvolution references may provide more accurate quantification of neuroinflammatory cell populations in PSCI. In addition, the M1/M2 polarization framework represents an oversimplified classification of microglial states. Microglia are increasingly recognized as exhibiting a dynamic spectrum of activation phenotypes rather than a strict binary pattern. Therefore, the M1‐like signatures reported here should be interpreted as reflecting a proinflammatory tendency rather than definitive subtype identity. Third, the present study was based on secondary analysis of public transcriptomic datasets and therefore cannot establish direct causal relationships between the identified hub genes and PSCI progression. Further experimental validation, including gene perturbation and pharmacological intervention studies, is still required. In addition, all current analyses were based on animal data, and the translational consistency between animal models and human PSCI remains to be established in future studies with larger cohorts, especially clinical samples.

## 5. Conclusion

In summary, by integrating bulk transcriptomic and single‐cell RNA‐seq data, this study identified LDHA, GSDMD, and CASP1 as core diagnostic genes associated with the lactate metabolism–pyroptosis axis in PSCI. These genes were further supported by cell–cell communication analysis linking metabolic dysregulation and neuroinflammatory signaling. The diagnostic model based on these three genes showed good performance in both the training and validation cohorts. In addition, drug prediction and molecular docking analyses nominated candidate compounds for future investigation. Overall, our findings support a potential role of the lactate metabolism–pyroptosis axis in PSCI and provide a basis for further mechanistic and translational studies.

## Author Contributions

Shulong Ge was responsible for conceptualization, methodology, formal analysis, and writing of the original draft. Qiying Zhang contributed to methodology, data curation, formal analysis, and drafting of the manuscript. Ning Liu participated in investigation, data curation, and validation. Xueyan Zheng was involved in software implementation, validation, and data visualization. Han Xu contributed to resources and investigation. Li Zhang conceived and supervised the study, managed project administration and funding acquisition, and critically reviewed and edited the manuscript. Shulong Ge and Qiying Zhang are co‐first authors and contributed equally to this work.

## Funding

This study was supported by Shandong Provincial Traditional Chinese Medicine Science and Technology Project (Grant No. Q‐2023065).

## Disclosure

All authors reviewed and approved the final version of the manuscript.

## Ethics Statement

This study was based exclusively on secondary analysis of publicly available transcriptomic datasets from the GEO database. No new human participants were recruited, and no new animal experiments were conducted by the authors. The original studies that generated these animal datasets were responsible for obtaining the relevant ethical approvals. Therefore, no additional ethical approval was required for the present study.

## Conflicts of Interest

The authors declare no conflicts of interest.

## Supporting Information

Additional supporting information can be found online in the Supporting Information section.

## Supporting information


**Supporting Information 1** Table S1: Datasets used in discovery and validation analyses.

**Supporting Information 2** Table S2: KEGG pathway enrichment results of DEGs in the discovery cohort.

**Supporting Information 3** Table S3: WGCNA module–trait relationships and key module summary.

**Supporting Information 4** Table S4: Hub genes selected by LASSO, SVM‐RFE, and random forest, with diagnostic performance.

**Supporting Information 5** Table S5: Candidate compounds from CMap and molecular docking results with hub genes.

**Supporting Information 6** Table S5B: Secondary candidate compounds identified by connectivity map (CMap) analysis with normalized connectivity scores between −0.85 and −0.90.

**Supporting Information 7** Table S6: Complete list of the 24 lactate metabolism– and pyroptosis‐related genes used for candidate gene construction.

**Supporting Information 8** Figure S1: MCP‐counter validation of CIBERSORT/LM22 immune deconvolution results.

**Supporting Information 9** Figure S2: Hub gene expression across all annotated cell types in scRNA‐seq data.

## Data Availability

The datasets analyzed in this study are publicly available in the Gene Expression Omnibus (GEO) repository under accession numbers GSE223580, GSE137482, and GSE174574. No new datasets were generated during this study.
