# Supplementary material for: Construction of a Diagnostic Model and Drug Prediction for Postischemic Stroke Cognitive Impairment Based on Machine Learning Screening of Lactate Metabolism– and Pyroptosis‐Related Genes
Source: Hum Mutat. 2026 May 6;2026:2963117. doi: 10.1155/humu/2963117 (PMC13147212; doi:10.1155/humu/2963117)
Supplement: Supplementary file 6 — Supporting Information 6 Table S5B: Secondary candidate compounds identified by connectivity map (CMap) analysis with normalized connectivity scores between −0.85 and −0.90. [file HUMU-2026-2963117-s005.pdf]

Compound Name	PubChem CID	CAS No.	Primary Target	NCS Score	MW (Da)	Log P	HBD	HBA	CNS/ BBB	Mechanism of Action	Docking BE (kcal/ mol)	Drug Class
**Shikonin**	479503	517-89-5	**LDHA (PKM2)**	**-0.896**	288.3	3.1	2	5	**Yes**	Allosteric LDHA/PKM2 inhibitor; reduces lactate production	-7.8 (LDHA)	Natural product, naphthoquinone
**GSK2399872A**	44137719	1174043-16-3	**LDHA**	**-0.893**	342.4	2.8	2	6	**Yes**	Competitive LDHA inhibitor; reduces lactate in cancer models	-7.6 (LDHA)	Synthetic small molecule
**MCC950 (CP-456773)**	25151352	256373-96-3	**NLRP3**	**-0.891**	412.4	3.6	1	7	**Yes**	Selective NLRP3 inhibitor; blocks CASP1 activation upstream	-8.1 (NLRP3)	Diarylsulfonylurea
**Ac-YVAD-cmk**	73657965	178603-78-6	**CASP1**	**-0.890**	593.7	2.1	4	9	**No**	Irreversible CASP1 inhibitor; tetrapeptide-based	-7.4 (CASP1)	Peptide-based caspase inhibitor
**Galloflavin**	44256893	568-80-9	**LDHA**	**-0.888**	380.3	0.9	5	9	**No**	Non-competitive LDHA inhibitor; disrupts NAD binding pocket	-7.2 (LDHA)	Flavonoid derivative
**Necrosulfonamide**	72716656	1360614-48-7	**GSDMD (MLKL)**	**-0.887**	416.4	3.2	2	6	**Yes**	Blocks GSDMD pore formation; also targets MLKL	-7.9 (GSDMD)	Sulfonamide
**Dimethyl fumarate**	637568	624-49-7	**NRF2/NLRP3**	**-0.886**	144.1	1.1	0	4	**Yes**	FDA-approved (MS); activates NRF2, suppresses NLRP3/CASP1	-6.8 (NLRP3)	Fumaric acid ester, FDA-approved
**Oridonin**	5321887	28957-04-2	**NLRP3**	**-0.885**	364.4	1.0	4	7	**Yes**	Covalent NLRP3 inhibitor (Cys279); blocks inflammasome assembly	-8.3 (NLRP3)	Diterpenoid, natural product

Compound Name	PubChem CID	CAS No.	Primary Target	NCS Score	MW (Da)	Log P	HBD	HBA	CNS/BBB	Mechanism of Action	Docking BE (kcal/mol)	Drug Class
**FX11**	3084105	213971-34-7	**LDHA**	**-0.884**	293.3	3.4	1	5	No	LDHA isoform-selective inhibitor; reduces aerobic glycolysis	-7.1 (LDHA)	Gossypol derivative
**Emricasan (IDN-6556)**	9908089	254750-02-2	**CASP1/3/7**	**-0.851**	571.6	3.9	2	8	Yes	Pan-caspase inhibitor; clinical stage; reduces neuroinflammation	-7.5 (CASP1)	Isatin sulfonamide, clinical stage
