# Supplementary material for: Construction of a Diagnostic Model and Drug Prediction for Postischemic Stroke Cognitive Impairment Based on Machine Learning Screening of Lactate Metabolism– and Pyroptosis‐Related Genes
Source: Hum Mutat. 2026 May 6;2026:2963117. doi: 10.1155/humu/2963117 (PMC13147212; doi:10.1155/humu/2963117)
Supplement: Supplementary file 7 — Supporting Information 7 Table S6: Complete list of the 24 lactate metabolism– and pyroptosis‐related genes used for candidate gene construction. [file HUMU-2026-2963117-s006.pdf]

Gene Symbol	Human EntrezID	Mouse Ortholog	Gene Category	MSigDB Gene Set	MSigDB ID	Regulation in PSCI (|logFC|>1, adj.p<0.05)	Biological Function in PSCI Context	Key Evidence in This Study	Supporting References
**LDHA**	3939	**Ldha**	**Lactate Metabolism**	HALLMARK_GLYCOLYSIS	**M5937**	Up (logFC=2.1, p<0.001)	Rate-limiting enzyme converting pyruvate to lactate; supports anaerobic glycolysis under ischemic hypoxia; regulates microglial M1/M2 polarization via lactate signaling	Highest expression in microglia (scRNA-seq); r>0.75 with M1 microglial infiltration; LDHA inhibition reduces infarct size in BCAS model	Xiong et al. 2024 (Theranostics); Liu et al. 2025 (Front Neurol); Jin et al. 2022 (Front Neurol)
**LDHB**	3945	**Ldhb**	**Lactate Metabolism**	HALLMARK_GLYCOLYSIS	**M5937**	Down (logFC=-1.3, p=0.004)	Catalyzes conversion of lactate back to pyruvate; LDHB downregulation shifts equilibrium toward lactate accumulation; LDHA/LDHB ratio determines net lactate flux	LDHA/LDHB imbalance reported in ischemic brain; LDHB loss promotes pro-inflammatory lactate accumulation	Liu et al. 2025 (Front Neurol); Wang et al. 2025 (Cell Death Dis)
**SLC16A1**	6566	**Slc16a1**	**Lactate Metabolism**	HALLMARK_GLYCOLYSIS	**M5937**	Up (logFC=1.5, p=0.002)	MCT1: monocarboxylate transporter 1; primary lactate export transporter in neurons and astrocytes; upregulation facilitates extracellular lactate accumulation	MCT1 upregulation documented in ischemic brain; facilitates astrocyte-to-neuron lactate shuttling	Xiong et al. 2024 (Theranostics); Liu et al. 2025 (Front Neurol)

Gene Symbol	Human EntrezID	Mouse Ortholog	Gene Category	MSigDB Gene Set	MSigDB ID	Regulation in PSCI (|logFC|>1, adj.p<0.05)	Biological Function in PSCI Context	Key Evidence in This Study	Supporting References
**SLC16A3**	9122	**Slc16a3**	**Lactate Metabolism**	HALLMARK_GLYCOLYSIS	**M5937**	Up (logFC=1.8, p=0.001)	MCT4: high-affinity lactate exporter; predominantly expressed in glycolytic cells including activated microglia; promotes lactate export under high glycolytic flux conditions	MCT4 upregulation in activated microglia; correlates with M1 polarization in PSCI model	Wang et al. 2025 (Cell Death Dis); Liu et al. 2025 (Front Neurol)
**PDK1**	5163	**Pdk1**	**Lactate Metabolism**	HALLMARK_GLYCOLYSIS	**M5937**	Up (logFC=1.4, p=0.008)	Pyruvate dehydrogenase kinase 1; phosphorylates/inactivates PDH complex, blocking pyruvate entry into TCA cycle and redirecting flux toward lactate production; induced by HIF-1alpha	PDK1 upregulation is a hallmark of Warburg-like metabolic shift in ischemic brain; targeted by dichloroacetate in stroke models	Liu et al. 2025 (Front Neurol); Wang et al. 2025 (Cell Death Dis)
**PKM2**	5315	**Pkm2**	**Lactate Metabolism**	HALLMARK_GLYCOLYSIS	**M5937**	Up (logFC=1.6, p=0.003)	Pyruvate kinase M2 isoform; rate-limiting glycolytic enzyme; PKM2 nuclear translocation promotes inflammatory gene expression in M1 microglia independent of its glycolytic function	PKM2 nuclear form promotes NLRP3-related gene transcription; pharmacological targeting with TEPP-46 reduces neuroinflammation	Liu et al. 2025 (Front Neurol); Sanman et al. 2016 (eLife)
**HK2**	3099	**Hk2**	**Lactate Metabolism**	HALLMARK_GLYCOLYSIS	**M5937**	Up (logFC=1.3, p=0.012)	Hexokinase 2: first committed step of glycolysis; HK2 upregulation is a hallmark of metabolic reprogramming; localizes to mitochondria and regulates NLRP3 priming	HK2 overexpression reported in activated microglia; HK2-NLRP3 interaction promotes inflammasome priming in glycolytic stress	Sanman et al. 2016 (eLife); Liu et al. 2025 (Front Neurol)
**GSDMD**	79792	**Gsdmd**	**Pyroptosis/Inflammasome**	REACTOME_PYROPTOSIS	**M27580**	Up (logFC=2.4, p<0.001)	Gasdermin D: pore-forming executor of pyroptosis; N-terminal domain (after CASP1/CASP4/5 cleavage) inserts into plasma membrane, causing lytic cell death and IL-1beta/IL-18 release	Hub gene (identified by LASSO+SVM-RFE+RF); AUC 0.795 (individual); positively correlated with CASP1; validated in MCAO cohort (p<0.0001)	Yu et al. 2021 (Signal Transduct Target Ther); Liu et al. 2024 (J Transl Med); Ge et al. 2024 (CNS Neurosci Ther)

Gene Symbol	Human EntrezID	Mouse Ortholog	Gene Category	MSigDB Gene Set	MSigDB ID	Regulation in PSCI (|logFC|>1, adj.p<0.05)	Biological Function in PSCI Context	Key Evidence in This Study	Supporting References
**GSDME**	1687	**Gsdme**	**Pyroptosis/Inflammasome**	REACTOME_PYROPTOSIS	**M27580**	Up (logFC=1.2, p=0.018)	Gasdermin E (DFNA5): alternative pyroptosis executor; cleaved by caspase-3; mediates secondary necrosis after apoptosis; expressed in neurons	GSDME-mediated neuronal pyroptosis reported in ischemic stroke; GSDME cleavage by CASP3 bridges apoptosis-pyroptosis continuum	Yu et al. 2021 (Signal Transduct Target Ther)
**CASP1**	834	**Casp1**	**Pyroptosis/Inflammasome**	REACTOME_PYROPTOSIS	**M27580**	Up (logFC=2.2, p<0.001)	Caspase-1: canonical inflammasome effector caspase; processes pro-IL-1beta/pro-IL-18 to mature forms; cleaves GSDMD to trigger pyroptotic pore formation; central to NLRP3 pathway	Hub gene (identified by LASSO+SVM-RFE+RF); AUC 0.805 (individual); IL1B-IL1R1 axis confirmed by CellChat; validated in MCAO cohort	Huang et al. 2021 (Cell Mol Immunol); Long et al. 2023 (Front Pharmacol); Liu et al. 2024 (J Transl Med)
**CASP4**	837	**Casp4**	**Pyroptosis/Inflammasome**	REACTOME_PYROPTOSIS	**M27580**	Up (logFC=1.1, p=0.028)	Caspase-4 (CASP11 in mouse): non-canonical inflammasome sensor for cytosolic LPS; directly cleaves GSDMD independently of NLRP3/CASP1; activated by mitochondrial stress	CASP4/11-GSDMD axis activated in ischemic brain; non-canonical pyroptosis contributes to post-stroke neuroinflammation	Yu et al. 2021 (Signal Transduct Target Ther)
**CASP5**	838	**Casp5**	**Pyroptosis/Inflammasome**	REACTOME_PYROPTOSIS	**M27580**	Up (logFC=0.9, p=0.041)	Caspase-5: non-canonical inflammasome caspase; cleaves GSDMD; cooperates with CASP4 in response to cytosolic LPS and mitochondrial DAMPs	CASP5 expression upregulated in PSCI discovery cohort; functional role in non-canonical pyroptosis in brain ischemia under investigation	Yu et al. 2021 (Signal Transduct Target Ther)
**NLRP3**	114548	**Nlrp3**	**Pyroptosis/Inflammasome**	REACTOME_PYROPTOSIS	**M27580**	Up (logFC=2.0, p<0.001)	NLR family pyrin domain-containing 3: canonical inflammasome sensor; assembles with ASC (PYCARD) and pro-CASP1 to form the NLRP3 inflammasome complex; activated by ATP, ROS, uric acid crystals, mitochondrial damage	Strongly upregulated in PSCI (volcano plot, Figure 1D); NLRP3 inhibition (MCC950, ChemR23 pathway) reduces PSCI severity; intersects with glycolytic flux via ROS	Long et al. 2023 (Front Pharmacol); Liu et al. 2024 (J Transl Med); Xu et al. 2025 (Cell Mol Immunol); Sanman et al. 2016 (eLife)

Gene Symbol	Human EntrezID	Mouse Ortholog	Gene Category	MSigDB Gene Set	MSigDB ID	Regulation in PSCI (|logFC|>1, adj.p<0.05)	Biological Function in PSCI Context	Key Evidence in This Study	Supporting References
**PYCARD**	29108	**Pycard**	**Pyroptosis/Inflammasome**	REACTOME_PYROPTOSIS	**M27580**	Up (logFC=1.3, p=0.011)	ASC (apoptosis-associated speck-like protein containing CARD): adaptor protein bridging NLRP3 to pro-CASP1; ASC speck formation is a hallmark of inflammasome activation; required for canonical NLRP3 inflammasome assembly	ASC speck formation observed in microglia of PSCI models; ASC inhibition reduces CASP1 activation and pyroptotic death	Huang et al. 2021 (Cell Mol Immunol); Long et al. 2023 (Front Pharmacol)
**AIM2**	9447	**Aim2**	**Pyroptosis/Inflammasome**	REACTOME_PYROPTOSIS	**M27580**	Up (logFC=1.1, p=0.025)	Absent in melanoma 2: cytosolic dsDNA sensor forming the AIM2 inflammasome; activated by mitochondrial DNA released during ischemic cell death; activates CASP1-GSDMD pathway independently of NLRP3	AIM2 activation by mitochondrial dsDNA release contributes to post-ischemic neuroinflammation; complements NLRP3 pathway	Long et al. 2023 (Front Pharmacol); Xu et al. 2025 (Cell Mol Immunol)
**IL1B**	3553	**Il1b**	**Pyroptosis/Inflammasome**	REACTOME_PYROPTOSIS	**M27580**	Up (logFC=2.3, p<0.001)	Interleukin-1 beta: key pyroptosis-released cytokine; processed from pro-form by CASP1; amplifies neuroinflammation through IL1R1 signaling on neurons and astrocytes; promotes M1 microglial polarization	Strongly upregulated in PSCI (Figure 1D); IL1B-IL1R1 identified as key microglia-neuron communication pair by CellChat (Figure 5F)	Huang et al. 2021 (Cell Mol Immunol); Xu et al. 2025 (Cell Mol Immunol)
**IL18**	3606	**Il18**	**Pyroptosis/Inflammasome**	REACTOME_PYROPTOSIS	**M27580**	Up (logFC=1.4, p=0.007)	Interleukin-18: pro-inflammatory cytokine released during pyroptosis; processed by CASP1; promotes NK cell activation, IFN-gamma secretion; contributes to post-stroke adaptive immune activation	IL18 upregulation in PSCI; plasma IL-18 levels associate with post-stroke cognitive outcomes in clinical studies	Huang et al. 2021 (Cell Mol Immunol); Long et al. 2023 (Front Pharmacol)
**HIF1A**	3091	**Hif1a**	**Cross-regulatory Node**	Literature-curated	**N/A**	Up (logFC=1.8, p=0.002)	Hypoxia-inducible factor 1-alpha: master transcriptional regulator of hypoxic response; upregulates LDHA, HK2, PDK1, SLC16A1 to promote anaerobic glycolysis; also promotes NLRP3 transcription under ischemia	HIF-1 signaling pathway enriched in KEGG analysis (Figure 1F); HIF1A-LDHA axis drives metabolic shift in ischemic microglia	Liu et al. 2025 (Front Neurol); Long et al. 2023 (Front Pharmacol); Wang et al. 2025 (Cell Death Dis)

Gene Symbol	Human Entrez ID	Mouse Ortholog	Gene Category	MSigDB Gene Set	MSigDB ID	Regulation in PSCI (|logFC|>1, adj.p<0.05)	Biological Function in PSCI Context	Key Evidence in This Study	Supporting References
**HMGB1**	3146	**Hmgb1**	**Cross-regulatory Node**	Literature-curated	**N/A**	Up (logFC=1.9, p=0.001)	High-mobility group box 1: damage-associated molecular pattern (DAMP); released during pyroptotic cell death; activates TLR4 and RAGE to amplify neuroinflammation; promotes NLRP3 priming signal	HMGB1 upregulated in PSCI volcano plot (Figure 1D); HMGB1-TLR4-NLRP3 axis represents key crosstalk node between lactate metabolism and pyroptosis	Long et al. 2023 (Front Pharmacol); Xu et al. 2025 (Cell Mol Immunol)
**TXNIP**	10628	**Txnip**	**Cross-regulatory Node**	Literature-curated	**N/A**	Up (logFC=1.6, p=0.004)	Thioredoxin-interacting protein: ROS sensor that activates NLRP3 when cellular oxidative stress exceeds thioredoxin scavenging capacity; bridges ROS accumulation (from glycolytic shift) to NLRP3 inflammasome activation; TXNIP upregulation under HIF-1alpha	TXNIP-NLRP3 pathway: identified in TLR4/NF-kB/NLRP3 signaling review; high glycolytic flux elevates ROS and TXNIP, connecting lactate metabolism to pyroptosis	Long et al. 2023 (Front Pharmacol); Sanman et al. 2016 (eLife); Wang et al. 2025 (Cell Death Dis)
**NFKB1**	4790	**Nfkb1**	**Cross-regulatory Node**	Literature-curated	**N/A**	Up (logFC=1.5, p=0.006)	Nuclear factor kappa B subunit 1: central transcription factor for inflammatory gene expression; required for NLRP3 transcriptional priming (signal 1); activated by TLR4/HMGB1; also promotes LDHA transcription in inflammatory conditions	NF-kB pathway activated downstream of TLR4/HMGB1 in PSCI; represents the signal-1 priming node for NLRP3 activation	Long et al. 2023 (Front Pharmacol); Xu et al. 2025 (Cell Mol Immunol)
**SIRT2**	22933	**Sirt2**	**Cross-regulatory Node**	Literature-curated	**N/A**	Down (logFC=-1.2, p=0.015)	Sirtuin 2: NAD+-dependent deacetylase; deacetylates and suppresses NLRP3 activity; also deacetylates LDHA to modulate its activity; SIRT2 downregulation under low NAD+ (due to high LDHA activity) promotes NLRP3 activation — representing a feedback loop between lactate metabolism and pyroptosis	SIRT2 downregulation creates permissive environment for NLRP3 hyperactivation; NAD+ depletion by LDHA activity links metabolic and inflammatory axes	Wang et al. 2025 (Cell Death Dis); Wang et al. 2025 (Cell Death Dis)

Gene Symbol	Human Entrez ID	Mouse Ortholog	Gene Category	MSigDB Gene Set	MSigDB ID	Regulation in PSCI (|logFC|>1, adj.p<0.05)	Biological Function in PSCI Context	Key Evidence in This Study	Supporting References
**STAT3**	6774	**Stat3**	**Cross-regulatory Node**	Literature-curated	**N/A**	Up (logFC=1.4, p=0.009)	Signal transducer and activator of transcription 3: activated by IL-6/IL-10 signaling; promotes M2 microglial polarization protective genes but also promotes astrocyte-mediated lactate production; STAT3-HIF1A cooperation amplifies glycolytic gene expression	STAT3 activation in reactive astrocytes promotes LDHA expression; STAT3-mediated regulation of microglial phenotype in post-stroke inflammation	Zeng et al. 2024 (Front Aging Neurosci); Wang et al. 2025 (Cell Death Dis)
**TLR4**	7099	**Tlr4**	**Cross-regulatory Node**	Literature-curated	**N/A**	Up (logFC=1.7, p=0.003)	Toll-like receptor 4: pattern recognition receptor for LPS and DAMPs including HMGB1; activates NF-kB (NLRP3 priming signal 1) and promotes glycolytic reprogramming via HIF-1alpha; TLR4/NF-kB/NLRP3 axis is a primary inflammatory signaling pathway in PSCI	TLR4 signaling enriched in KEGG analysis (Toll-like receptor pathway, Figure 1F); TLR4/NF-kB/NLRP3/CASP1/GSDMD cascade directly links DAMPs to pyroptotic execution	Long et al. 2023 (Front Pharmacol); Xu et al. 2025 (Cell Mol Immunol)
