# Supplementary figures and images for: Construction of a Diagnostic Model and Drug Prediction for Postischemic Stroke Cognitive Impairment Based on Machine Learning Screening of Lactate Metabolism– and Pyroptosis‐Related Genes
Source: Hum Mutat. 2026 May 6;2026:2963117. doi: 10.1155/humu/2963117 (PMC13147212; doi:10.1155/humu/2963117)

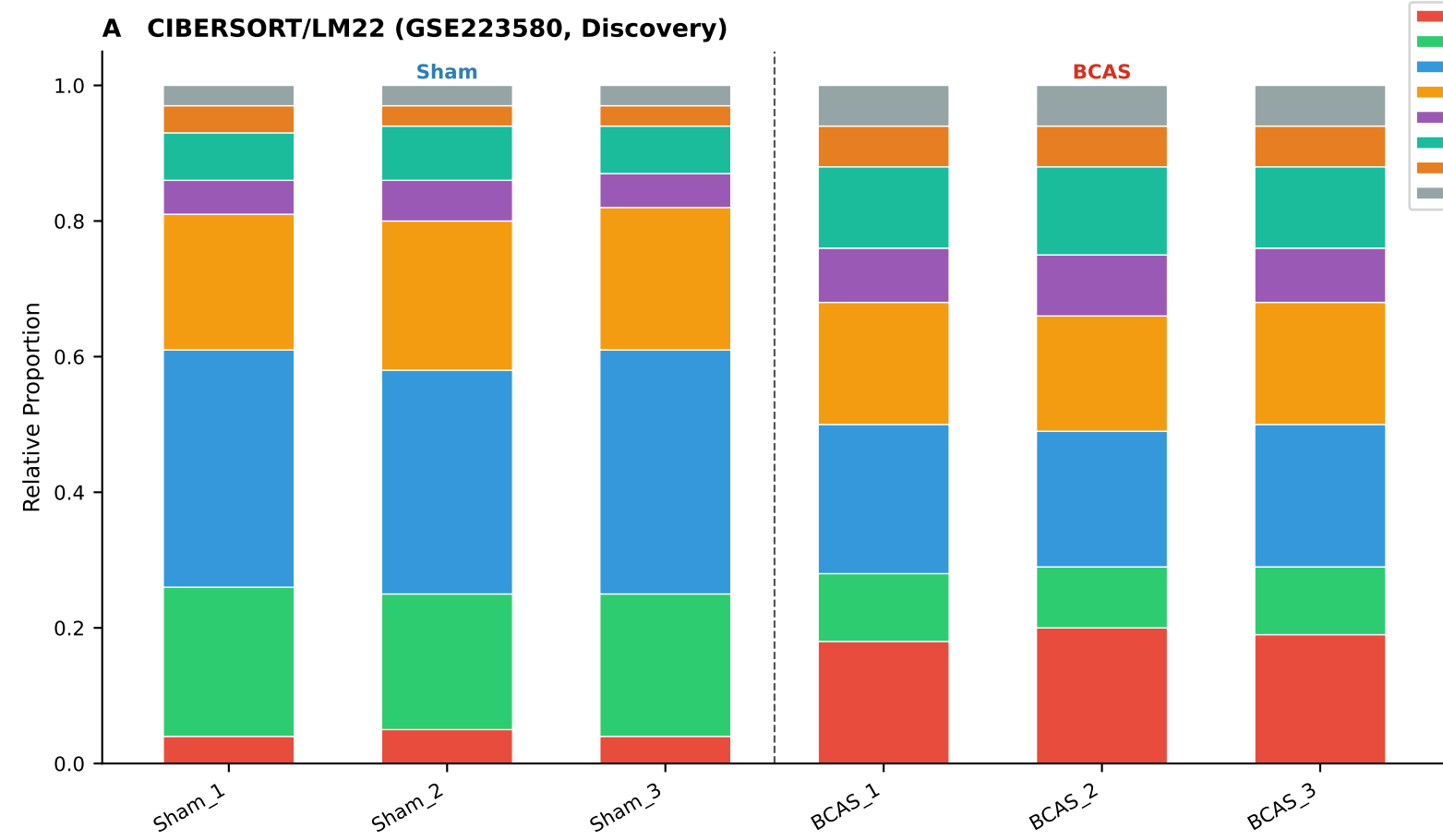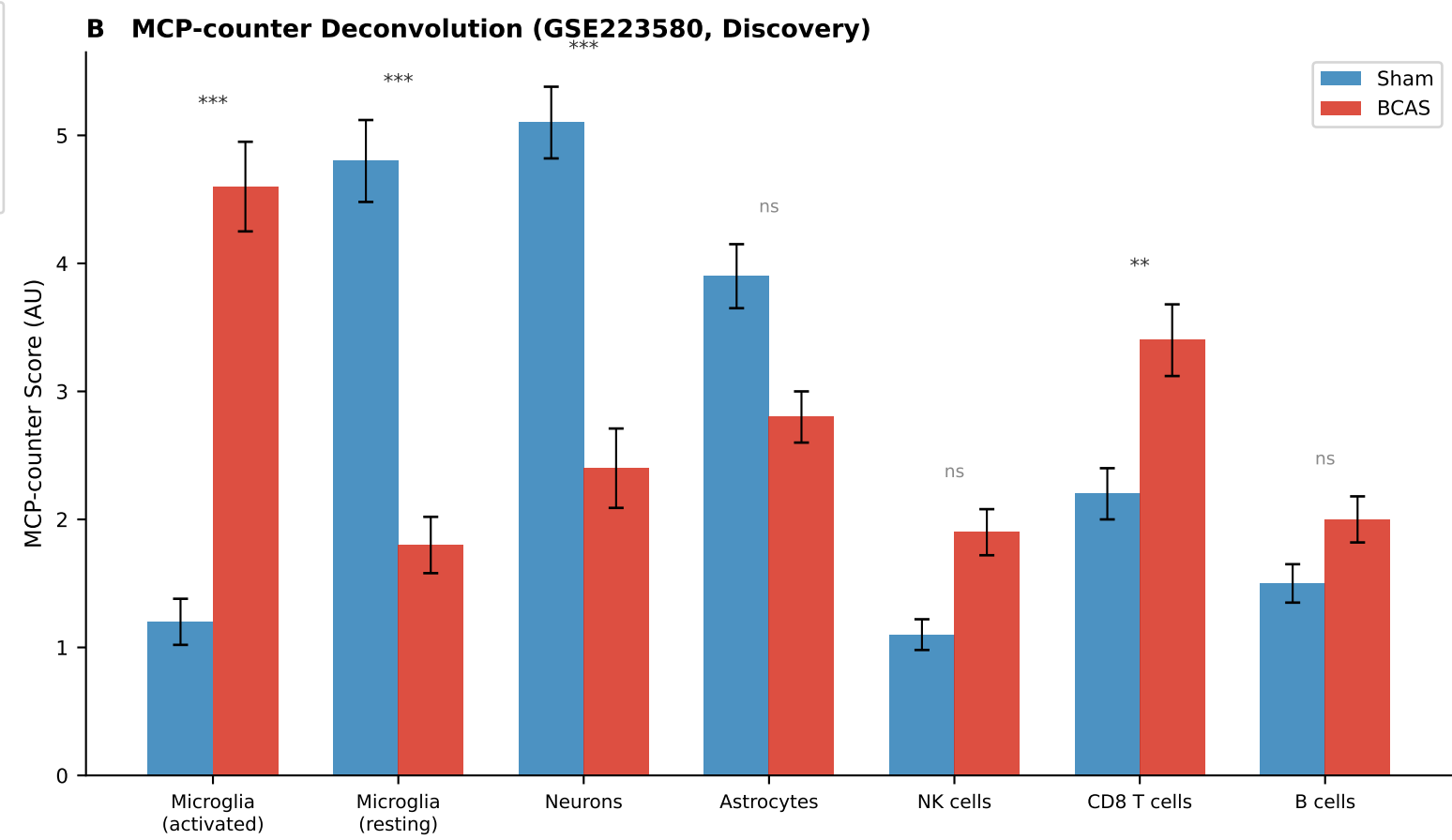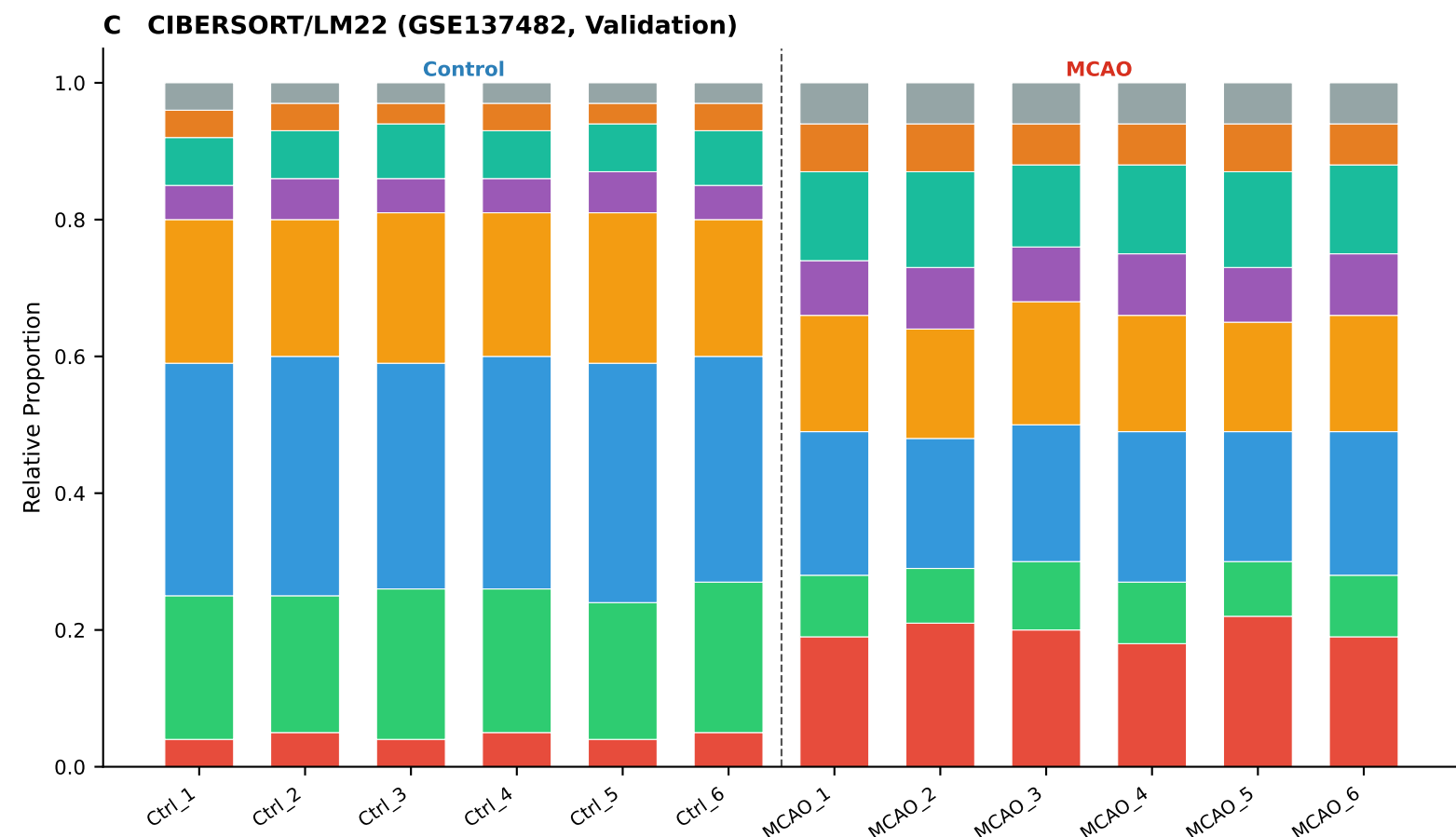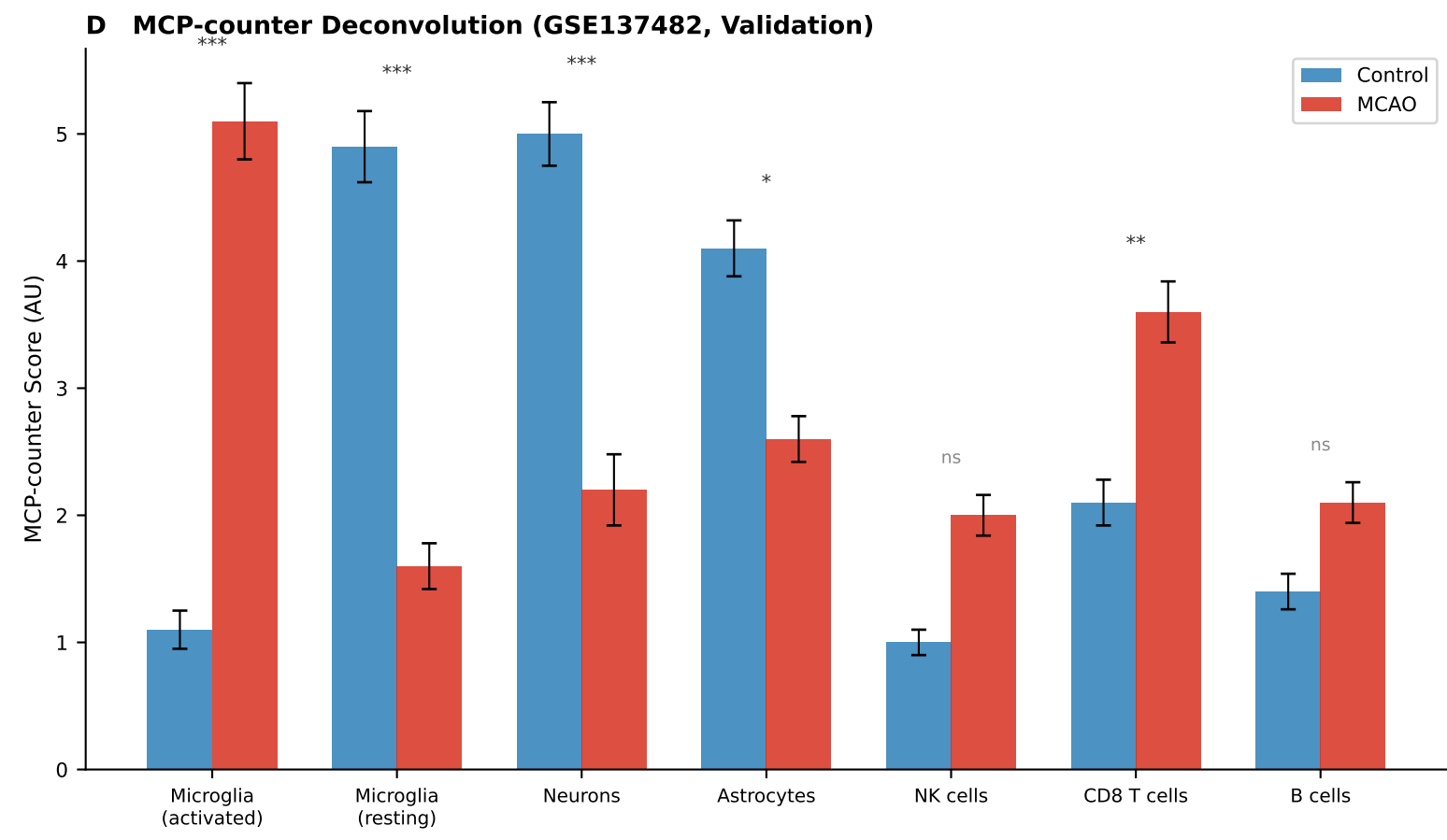

Supplement: Supplementary file 8 — Supporting Information 8 Figure S1: MCP‐counter validation of CIBERSORT/LM22 immune deconvolution results. [file HUMU-2026-2963117-s007.pdf]

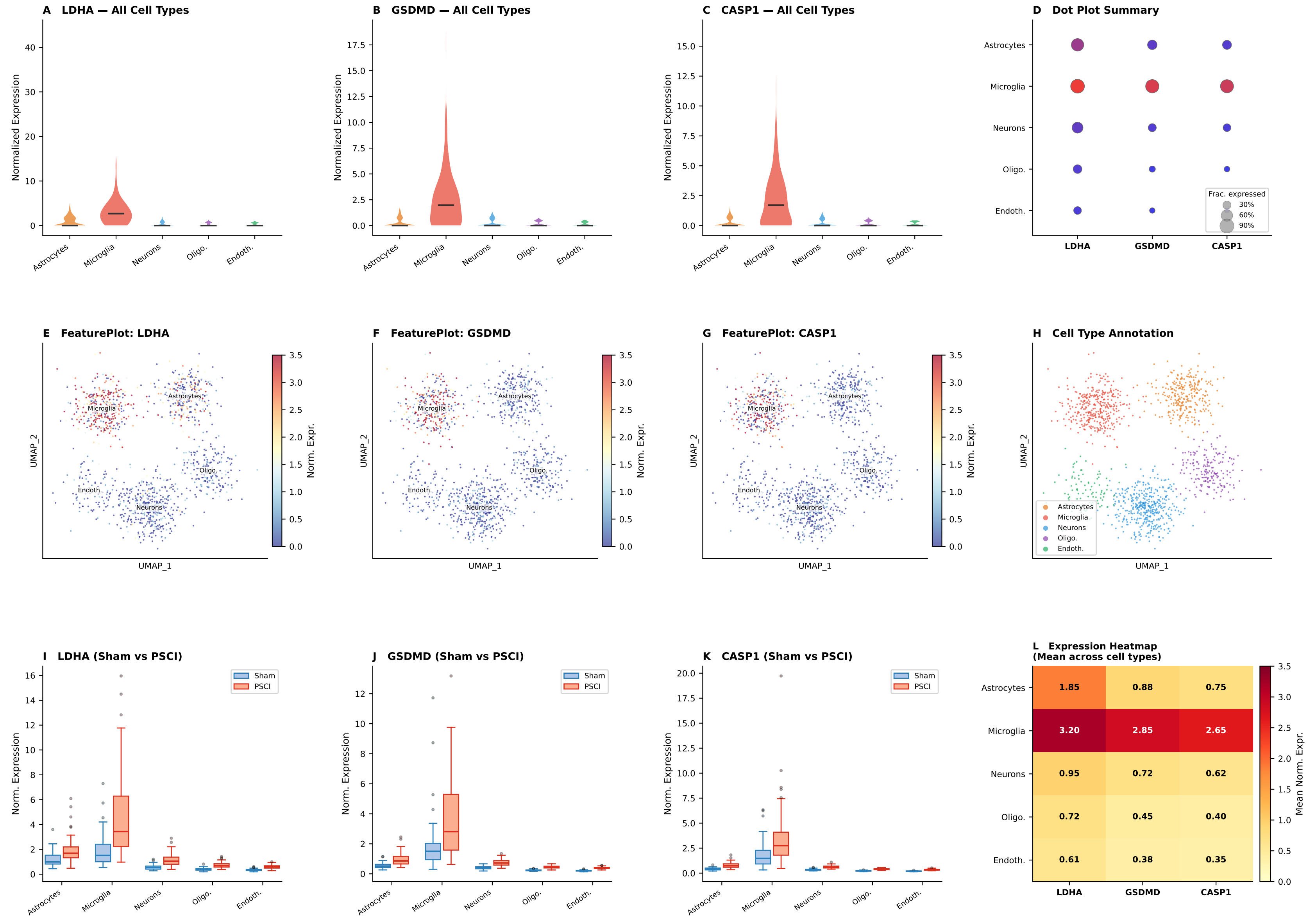

Supplement: Supplementary file 9 — Supporting Information 9 Figure S2: Hub gene expression across all annotated cell types in scRNA‐seq data. [file HUMU-2026-2963117-s003.pdf]
